# ArtEMon: Artificial Intelligence and Internet of Things Powered Greenhouse Gas Sensing for Real-Time Emissions Monitoring

**DOI:** 10.3390/s23187971

**Published:** 2023-09-19

**Authors:** Ali Yavari, Irfan Baig Mirza, Hamid Bagha, Harindu Korala, Hussein Dia, Paul Scifleet, Jason Sargent, Caroline Tjung, Mahnaz Shafiei

**Affiliations:** 1 School of Science, Computing and Engineering Technologies, Swinburne University of Technology, Melbourne, VIC 3122, Australia; imirza@swin.edu.au; 2 6G Research and Innovation Lab, Swinburne University of Technology, Melbourne, VIC 3122, Australia; 3 Department of Infrastructure Engineering, University of Melbourne, Melbourne, VIC 3010, Australia; baghah@unimelb.edu.au; 4 Institute of Railway Technology, Monash University, Melbourne, VIC 3800, Australia; harindu.korala@monash.edu; 5 Department of Civil and Construction Engineering, Swinburne University of Technology, Melbourne, VIC 3122, Australia; hdia@swin.edu.au; 6 School of Business, Law and Entrepreneurship, Swinburne University of Technology, Melbourne, VIC 3122, Australiajpsargent@swin.edu.au (J.S.); 7 School of Design and Architecture, Swinburne University of Technology, Melbourne, VIC 3122, Australia; ctjung@swin.edu.au

**Keywords:** IoT, greenhouse gas, sustainable logistics, emissions, supply chain, AI

## Abstract

Greenhouse gas (GHG) emissions reporting and sustainability are increasingly important for businesses around the world. Yet the lack of a single standardised method of measurement, when coupled with an inability to understand the true state of emissions in complex logistics activities, presents enormous barriers for businesses to understanding the extent of their emissions footprint. One of the traditional approaches to accurately capturing and monitoring gas emissions in logistics is through using gas sensors. However, connecting, maintaining, and operating gas sensors on moving vehicles in different road and weather conditions is a large and costly challenge. This paper presents the development and evaluation of a reliable and accurate sensing technique for GHG emissions collection (or monitoring) in real-time, employing the Internet of Things (IoT) and Artificial Intelligence (AI) to eliminate or reduce the usage of gas sensors, using reliable and cost-effective solutions.

## 1. Introduction

Transport activity accounts for around one fifth of global carbon dioxide (CO${}_{2}$) emissions. Freight road activity accounts for around 29.4% of all transport emissions [1]. Demand for freight is expected to triple by 2050 compared to 2015, according to the International Transport Forum [2], fuelled by global supply chains, burgeoning economies in the developing world, and a rise in e-commerce activities.

In Australia, transport is the third-largest source of local greenhouse gases, accounting for 18.7% of all national emissions in 2022 [3]. The transport sector is also one of the strongest contributors to emissions growth in Australia. Emissions from transport have increased by nearly 60% since 1990—more than any other sector in the economy [3]. Within transport, road freight and supply chains present challenges to successful emissions reduction. Freight transport is a significant contributor to the sector’s emissions, and it is expected to grow as a proportion of total emissions [3].

Transport is facing growing pressure from regulators, financiers, and consumers to accelerate the transition to zero emissions. To enable the required reductions in the use of fossil fuels in the transportation sector, the sector will need to undergo a profound transformation in its energy use and the ability to accurately measure emissions from fossil fuels. There is a growing focus on the logistics emissions reporting function, as companies seek to uncover one of the least-known yet potentially highly carbon-intensive parts of their organisation’s operations. The granularity and accuracy of reporting is critical for the advancement of future states of logistics emissions management. Whilst many companies are still grappling with the backward-looking reporting task, to make a meaningful impact in logistics emissions, there needs to be a transition to forward-looking and performance monitoring of logistics emissions. Transport sector emissions analysis has been the focal point of numerous researches for many years. The studies show that there are multiple factors—including weather, road condition, vehicle condition, vehicle weight, and driving behaviour—that have significant impacts on vehicle emissions [4,5,6,7,8,9,10].

Analysis of vehicle emissions is normally conducted in laboratory environments, on a chassis dynamometer [11,12]. However, the studies show that there is a significant difference between data captured in a laboratory environment and data captured on roads [12,13].

Although laboratory analysis can capture useful data regarding vehicle emissions, it is almost impractical to evaluate all the real-world metrics that impact vehicle emissions in a laboratory environment. Laboratory emissions analysis has been found to underestimate emissions rates by 10–20% [11]. As a result, many researchers tend to install portable exhaust gas analysis devices on vehicles, to capture on-road data. However, installing and capturing on-road data can pose significant challenges.

The accuracy of these devices for long-term data collection is one of the challenges, because they need calibration before each measurement. Exhaust gas analysers are analytical devices that measure the concentration of a specific gas within a mixture of other gases. These devices typically comprise different gas sensors, using industry-standard non-dispersive infrared (NDIR) and chemical sensor technologies. NDIR technology measures the concentration of a gas, by determining how much infrared energy is absorbed at a select wavelength band that corresponds to a resonant mode spectrum of the analysed gas molecule. The chemical sensors technologies used in the analyser are usually based on electrochemical and metal-oxide semiconducting sensors, which suffer from long-term stability, selectivity, and fast response and recovery. The electrochemical sensors require regular replacement of electrolytes; therefore, the short life of these chemical sensors would add to the cost of experiments.

We previously conducted research in developing and installing an Internet of Things (IoT)-based platform, called ParcEMon [14], to capture live data from a delivery van, so as to analyse parcel-level emissions. During this research, multiple challenges were faced, in retrieving continuous data from the delivery van by using a gas analyser comprising different gas sensors, so as to measure the concentration of carbon monoxide (CO), carbon dioxide (CO${}_{2}$), hydrocarbons (HC), oxygen (O${}_{2}$), and nitrogen oxide (NO${}_{x}$) gases, as shown in Table 1.

One of the main challenges was installation of the gas analyser in a secure location, to be able to collect and analyse the gas flow without being impacted by vehicle movement and road condition in a real world context.

Since the gas analyser control unit was required to be close and connected to the gas flow, it could not be installed inside the cabin, as it could pose safety issues to the driver and other passengers. Therefore, the gas analyser was secured outside the car. However, there are multiple factors that can impact the possibility of installing the gas analyser and related accessories outside the car.

The first issue is that a vehicle’s sudden movements and vibrations can loosen the different components that transfer the gas flow to the gas analyser, including the exhaust nuzzle and gas pipe. As a result, all these components must be firmly installed, to withstand the vehicle movements and vibrations. In addition, a change in road conditions can also impact the gas analyser functionality. Bumpy roads can cause road surface contact with the devices and stop or suspend the sensors’ functionality. Moreover, dirt roads can cause gravel to hit the analyser or can cause mud splash on the analyser, which can cause full or partial blockage of gas flow, thus impacting the data quality. Moreover, rainy weather can also cause water splash on the analyser, which can damage the sensors and impact their functionality. Furthermore, installing the analyser in a sealed enclosure is also not practical, because there must be airflow, so as to avoid concentrated gas around the gas analyser, which can result in false data readings. Another issue regarding analyser installation is the high degree of heat emitted from the exhaust. This can damage the analyser pipe and sensors and cause data loss. A further challenge, regarding utilisation of a gas analyser in live data collection, is that the sensors normally require periodic checks, to ensure data quality and the functionality of the sensors. For example, one of the components of such gas analysers, which is connected to the exhaust nuzzle, is a water filter. This filter is required to reduce the moisture entering the gas analyser unit. After some hours of driving, and also depending on the weather conditions, this filter must be unplugged, to empty the water accumulated inside it, because the moisture that enters the gas analyser can impact the sensors’ accuracy and data quality. Moreover, the sensors require to be periodically restarted (i.e., zeroing) and calibrated, to improve measurement accuracy. Such periodic check-up requirements hinder the usage of such sensors for long-term data collection in a real world context.

Having discussed the challenges of deployment of a gas analyser to collect live data from on-road vehicles, investigation of alternative solutions to analysing vehicle emissions without employing gas sensors was a potential research area. These challenges could be addressed by using artificial intelligence (AI) techniques to estimate vehicle emissions based on vehicle data, such as speed, revolutions per minute (RPM), acceleration, and deceleration. By employing the dataset that has been collected using a gas analyser, the correlation between driver behaviour and vehicle emissions could be determined. The obtained dataset could then be used as a training set, to predict vehicle emissions in the long run, based on driver behaviour without a gas analyser. Such an approach could help to eliminate the challenge of installing a gas analyser and, at the same time, estimating the impact of driving behaviour on vehicle emissions.

In this research, the dataset collected, using a physical gas analyser and ParcEMon platform [14], was used to develop the training set, as well as to analyse the accuracy and validity of the machine learning technique, in predicting vehicle emissions.

This paper presents an AI-enabled emissions monitoring technique (referred as ArtEMon) that aims to utilise AI to replace a physical gas analyser attached to the exhausts and to produce accurate emissions reporting. The main contributions of this paper are as follows:

- An effective technique to align and merge the measurements from various IoT data sources, through a series of methods, from processing raw data to predictive emissions modelling using information from on-board diagnostics (OBDs), temperature, and humidity sensors.
- A weighted ensemble model that assigns weights based on the performance of the tree-based and stacked models, to estimate CO${}_{2}$ emissions.
- A feasibility study of our solution, through an experimental approach to evaluating how we predict emissions from the IoT data, without using a gas analyser.

The remainder of this paper is organised as follows. Section 2 describes three emissions reporting frameworks, as well as their comparison. Section 3 describes the architecture and methodology of the ArtEMon. Section 4 and Section 5 describe the IoT data collection, preprocessing, and the performance of our emissions prediction. Finally, Section 6 and Section 7 describe the discussion, future work, and conclusion of this paper.

## 2. Greenhouse Gas Emissions Reporting Frameworks

Corporate monitoring and reporting of Scope 3 GHG emissions [15] from the ’last-mile’ in the up-and-downstream transportation of goods services has been prioritised by governments and companies since the Paris Agreement of 2015 [16], with international and regional frameworks developed on the foundations of the Greenhouse Gas Protocol [17] initiative now in use.

The GHG Protocol Corporate Standard groups a company’s GHG emissions into three categories, from Scope 1 to Scope 3: Scope 1 refers to direct emissions; Scope 2 refers to indirect emissions and represents the emissions resulting from the generation of purchased energy (e.g., grid-supplied electricity); Scope 3 covers all the other indirect emissions in the company’s value chain that are not included in Scope 2 [17,18].

Scope 3 emissions can constitute a significant part of a company’s overall GHG footprint [19]. With estimates placed as high as 75% of all emissions for many companies [20], it is not surprising that the value of modelling, working with, and reporting Scope 3 emissions data is being recognised. Emissions data can be used to support investment, procurement, and sales strategies, by assessing the impact of various freight scenarios and predicting carbon return on investments. It provides a metric that allows companies to identify inefficiencies in their logistics network and work towards improved efficiencies. Companies can track progress towards climate goals and demonstrate corporate social responsibility, including positive climate and health impacts [21].

However, managing the collection and analysis of GHG emissions data remains a complex and frustrating challenge for companies. Current, local, and real-time data that account for the emissions produced in the delivery of domestic goods and services (whether by truck or freight consignment, courier, express post and parcel, or public transport networks) are not readily available data that are accessible for use. Current reporting is based on applying one of a range of available calculators, to estimate GHG emissions by measuring the distance a parcel travels and the fuel expended over the distance. In such approaches, there are many variables that cannot be easily accounted for, such as speed, road incline, and driver behaviour.

For companies to report Scope 3 emissions, they must apply the methodology from one of several key frameworks available for use, developed by different industry consortia in different regions and contexts. These frameworks include the Carbon Disclosure Project (CDP) [22], the EconTransistIT methodology [23], the GHG Protocol [17], the Global Logistics Emissions Council (GLEC) European framework [24], the Taskforce on climate-related financial disclosures [25], the USA’s Environmental Protection Agency’s Smartway Program [26], and the Australian National Greenhouse Energy Reporting Scheme [3]. More recently the International Standards Organisation, have published ISO 14083 [27], "Greenhouse gas management and related activities—Quantification and reporting of greenhouse gas emissions of transport operations", with the aim of providing a common methodology for the quantification and reporting of greenhouse gas emissions; however, its use remains voluntary.

**Figure 1:**
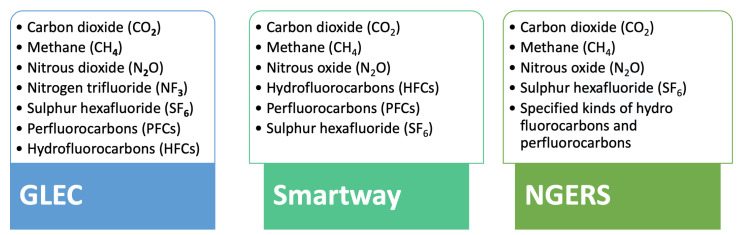
Gases included in the compared frameworks [24,26,28].

**Figure 2:**
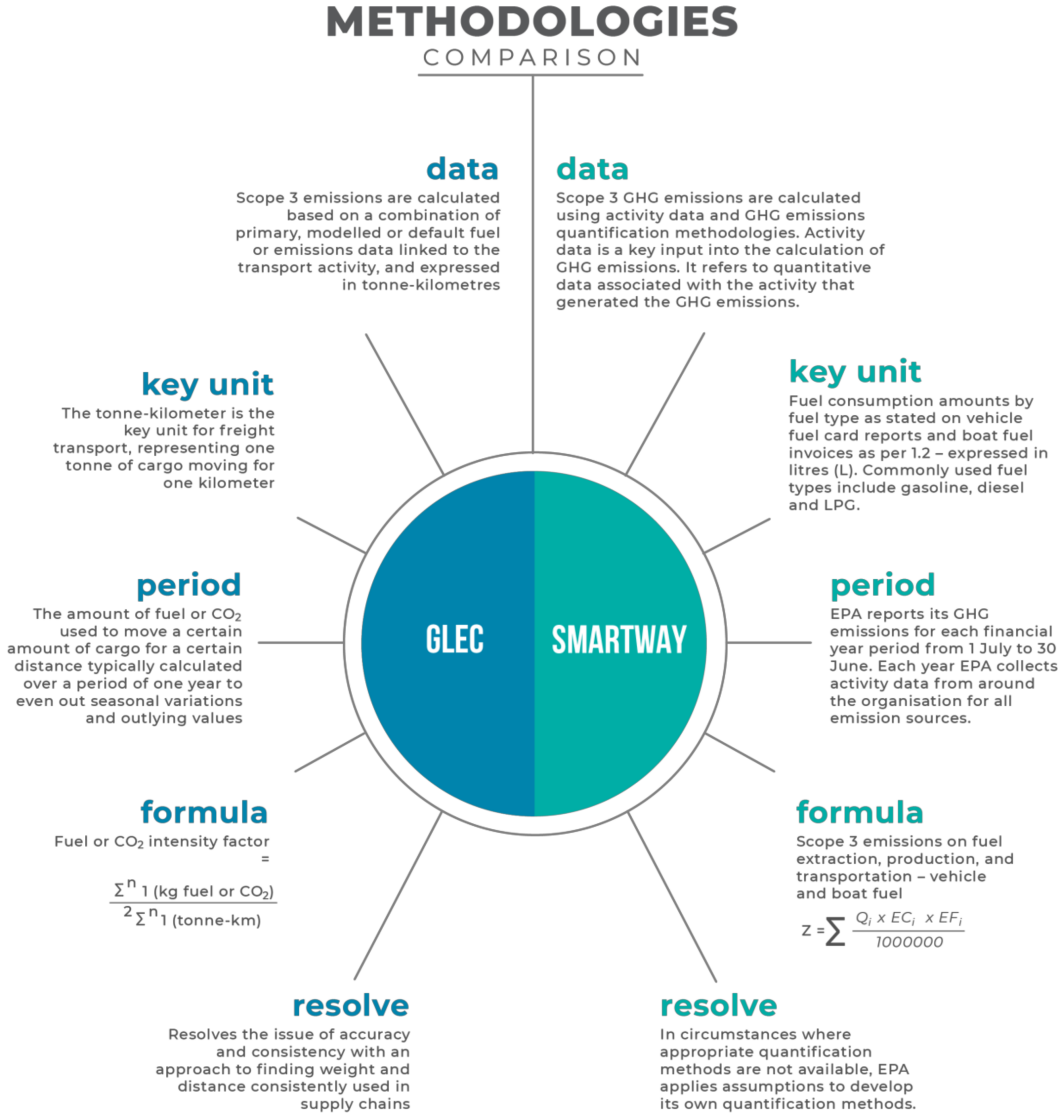
GHG reporting frameworks comparison.

For this project, the research team evaluated three of the leading regional frameworks, to develop an understanding of their use, the types of data collected, approaches to data collection, methodologies for calculating Scope 3 emissions, and granularity of reporting. These frameworks include GLEC [24], Smartway [26], and NGERS [28]. The objectives and approaches of the three frameworks are informed by and inform one other, but there are significant differences in their methodologies. Each framework varies in its approach to the measurement of GHG emissions and gases prioritisation (see Figure 1). While NGERS [28] establishes goals and specifies gases for emissions reporting, it does not present a methodology for Scope 3 data collection at all. Therefore, further evaluation has focused on a comparison of the GLEC [24] and Smartway [26] frameworks, including consideration of how each works with different calculation tools, different decision trees, and sets of historic data made available (See Figure 2). Each framework applies different assumptions about routes and different approaches to the measurement of fuel consumption per gram (FPG), and each varies in the range of gases reported [24,26,28].

For Scope 3 emissions, the absence of a single standardised method for measuring and modelling emissions across complex logistics activities, compounded by significant issues in sourcing and collecting accurate and timely data for reporting, presents enormous barriers for companies. There is an uncertainty about the accuracy of reported data, and about their ability to pursue the most cost-effective carbon mitigation strategies. This is exacerbated in Australia, where—under the National Greenhouse and Energy Reporting Act [29]—GHG emissions reporting is not yet mandatory [3], and the sheer complexity of data collection and reporting means that many who could participate voluntarily and benefit from more effective and efficient close-to-real-time reporting, are simply not participating.

## 3. Architecture and Methodology

The main objective of the research reported in this paper was to develop a complete end-to-end IoT-and-AI-enabled solution for monitoring GHG emissions from combustion engine vehicles, without relying on exhaust gas analysers. The IoT is defined as connecting a countless number of sensors and various devices (referred to as ’things’) through the internet, paving the way for innovative services and products [30].

**Figure 3:**
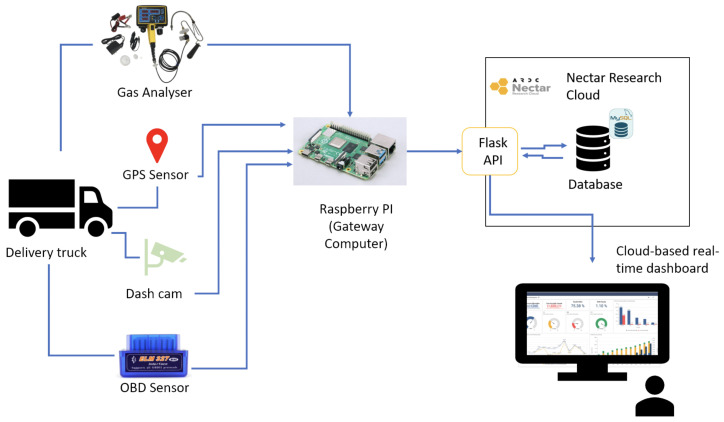
IoT -based testbed architecture.

Figure 3 illustrates the testbed architecture used to collect the raw data employed in this research. The development of this testbed was presented in [14]. The sensor data were collected from four different types of sensors, as follows:

- Gas Analyser (5 Gases): The analyser measured the concentration of carbon monoxide (CO), carbon dioxide (CO${}_{2}$), fuel-dependant hydrocarbons (HC), oxygen (O${}_{2}$), and nitrogen oxide (NO${}_{x}$) gases emitted from the exhaust of a delivery van. The data collected from the gas analyser were used to train and validate the AI models.
- OBDs: vehicle on-board diagnostics data were collected from the instrumented vehicle, to be used by the AI models. An OBD2 scanner provided comprehensive access to live and recorded data from the vehicle, including vehicle speed, acceleration, deceleration, and engine and fuel system operating conditions. These data were required, to correlate driving conditions to the emissions recorded by the gas analyser. OBDs sensory data are easy to collect, and are available from the majority of new cars produced over the past several years.
- Temperature and humidity sensor: the data related to the ambient condition of the delivery van were collected, using a temperature and humidity sensor located underneath the van.

The IoT sensor node included a small single-board computer (i.e., a Raspberry Pi) that was connected to the reporting gas analyser and OBD2 port through Bluetooth. The gas analyser collected the emissions data in real time and sent it to the computer through a serial cable. The data streams were annotated and synchronised in the computer and sent to the cloud through a broadband internet connection. This IoT sensor node was also connected to a dashcam and GPS module, to track the location of the vehicle and its speed and acceleration. A power bank was included, to provide the required power for running the IoT sensor node. In addition, the implemented enclosure included an automobile auxiliary power outlet for charging the power bank. Finally, a cooling system and environmental monitoring sensors (i.e., temperature and humidity) were deployed, to monitor the ambient temperature and humidity and to maintain the temperature inside the IoT sensor node enclosure. A detailed description of the testbed is described in [14].

Field testing was undertaken with a diesel delivery van in Vermont South in Victoria, Australia. The field trial was conducted under typical ’everyday’ or naturalistic driving conditions, employing a driver who regularly drove the vehicle. The vehicle was monitored while making its usual deliveries under normal driving conditions on urban roads, major arterial roads, and freeways. This included regular stop-and-go traffic, acceleration, deceleration, and smooth uniform driving during the trial.

**Figure 4:**
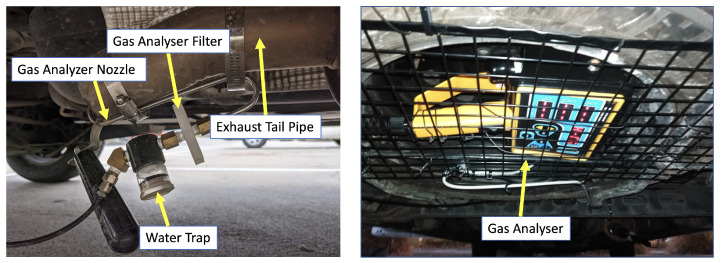
Gas analyser installed under the van.

The gas analyser was installed in the carriage, close to the exhaust, without interfering with driving conditions or safety, allowing for the collection of data under the ordinary driving conditions experienced by the driver, as illustrated in Figure 4.

The primary sensors used for the data collection included a gas analyser, vehicle onboard diagnostics (OBDs), and environmental condition sensors (i.e., humidity and temperature sensors). The data collected from these sensors were heterogeneous and contained inherent noise. These heterogeneous data were then processed, to remove noise and to identify relevant features. The data collected from the sensors were stored on the computer, using the JSON format. The first step involved reading the JSON files containing the sensor measurements and converting the raw timestamp (i.e., UNIX time) to a valid date–time format, for time-based data reporting. The timestamp was used as the main parameter, to perform the fusion of the heterogeneous data collected from various different sensors.

## 4. IoT Sensory Data Preprocessing

This section presents a discussion on the processing of the IoT sensory data. The first step involved reading the sensor data measurements and converting the raw timestamp to a valid date–time format. This produced 43,782 (gas analyser, including five gases), 14,562 (OBDs, including RPM, speed, FPG, etc.), and 192,972 (temperature and humidity) data points on the first day of the experiment.

### 4.1. Noise Removal

The next part of the IoT data processing involved identifying and removing incorrect sensor measurements. Most of the incorrect measurements were from the gas analyser. These measurements contained incorrect characters or partial values. Such measurements were considered as noise and were dropped from the analysis. There were several reasons for these noises, such as data loss because of communication issues, as the gas analyser was using serial protocol to transfer the data, using a long cable.

After removing all the rows containing the incorrect values, a total of 40,894 valid data points from the gas sensor measurements were used in the analysis.

### 4.2. Zeroing Process

Gas analysers need to purge the gas and perform calibration, based on the reference gases in the ambient air, frequently, to provide accurate readings. The gas analyser we used was set to purge the gas and perform the calibration process every 10 min.

We flagged the readings between the start-zeroing and finish-zeroing intervals. For that duration, the gas reading were not valid and were removed from the dataset, accordingly.

### 4.3. Lag Identification

**Figure 5:**
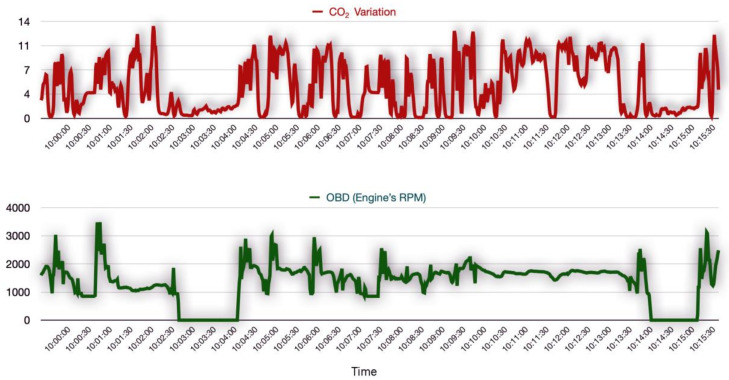
Identifying delay in gas analyser measurements.

There is usually a delay associated with gas analyser measurements when compared to the OBDs. This is because the gas concentration is measured by the gas analyser at the end of the exhaust pipe. The gas must travel through the exhaust pipe to reach the gas analyser, where it is sensed, and this normally takes a few seconds. The challenge here was to identify the right amount of delay and then offset the gas analyser measurements, to adjust the delay and align them with measurements from the other sensors. All the sensor measurements were first indexed based on their timestamp, and the measurements were re-sampled to the nearest one second of the time series, because the sensors reported measurements at millisecond granularity. Re-sampling usually introduces *null* values at the timestamps that were not in the original time series. Imputation is an important data preprocessing step for handling incomplete data, as the machine learning models for analysing IoT data from heterogeneous sensors commonly assume that the sensor data is complete. Such modelling results, which include missing or incomplete data, may be inaccurate and unreliable. It was, therefore, important to interpolate the missing values, by using the mean value of the respective features. Then, the used RPM measurements from the OBDs were used as a reference timestamp and were compared to the CO${}_{2}$ measurements from the gas analyser at various time intervals, to visually study the lag. As seen in Figure 5, the visual analysis of the time series helped to identify the delay, which was found to be approximately 6 s.

### 4.4. Offsetting, Re-Sampling, and Interpolating Measurements

To align the gas analyser measurements with the rest of the sensor measurements, the gas analyser measurements were offset by around 6 s and were aligned with the rest of the sensor measurements. IoT sensors do not necessarily produce data at regular intervals, and the IoT time series is highly irregular, with regard to its sampling rate, which makes the processing and analysis of such data challenging—specifically, when the data are received independently from multiple sensors, while they are being processed and analysed together. This makes the re-sampling of such irregular and unevenly spaced time series to a more regular and consistent frequency an important part of the IoT data processing step. To obtain a refined set of measurements, all data received from the gas analyser, OBDs, and temperature sensors were interpolated with their mean values at every one-second interval.

After performing interpolation on the data with the mean values of individual features, to fill the time points where there were no measurements, the next step was to align all measurements that had a common start and end time, because the starting and ending times of measurements are different for all sensors. This was required, to eliminate any missing values for the time series that ended early.

Table 2 identifies the starting and ending times of all the IoT sensors for the first day of the experiment. After re-sampling for every one second, interpolating, and aligning the measurements, a total of 15,778 data points were obtained from the gas, OBDs, temperature, and humidity sensors on a single day. All measurements from the sensors where the corresponding speed was reported as zero were also excluded. This allowed us to have a uniform time series for all measurements.

### 4.5. Relevant Feature Selection

To estimate the CO${}_{2}$ emissions, various features of the vehicles—such as speed, RPM, coolant temperature, acceleration, and throttle—were used [31,32,33]. Singh et al. [33] identified that speed and acceleration are highly correlated with CO${}_{2}$ emissions; however, these features on their own provide limited support in estimating CO${}_{2}$ emissions.

As seen in Algorithm 1, the merged measurements from all the sensors in this research (the OBDs sensors combined with the gas, temperature, and humidity sensors) were used to construct a dataset for understanding CO${}_{2}$ emissions. The final feature set, presented in Table 3, included information on the RPM, FPG, speed, temperature, and humidity, and the target feature was CO${}_{2}$.
**Algorithm** **1:** IoT sensor data preprocessing.Input: sensor measurements from gas analyser (*A*), OBDs (*B*), temperature and humidity (*C*)Output: modelling dataset *E*for each measurement in *A* do   remove noise   perform zeroing processend forfor each measurement in *A* do   offset ∀ measurement by 6 send forfor all measurements in *A*,*B*,*C* do   re-sample ∀ 1 s   interpolate with μ values   align timestampsend forConstruct modelling dataset E by merging *A*,*B*,*C*

## 5. Predicting CO${}_{2}$ Emissions

Ensemble learning is a class of machine learning technique that works by utilising multiple base learners that collectively improve the predictive performance. LightGBM [34], gradient boosting [35], and xGBoost [36] are some of the popular algorithms that have been used for regression and classification applications. In this paper, various gradient-boosting decision tree algorithms were used, including gradient boosting [35], xGBoost [36], and LightGBM [34]. Gradient-boosting decision trees are an ensemble of decision trees that use the best split points to learn the decision trees, make predictions on each decision tree, and then combine the individual predictions with those of other trees in the ensemble, to produce a strong prediction. Gradient boosting works by optimising the loss function; it sequentially tries to find new weak learners, based on the residuals from the previous weak learners in each iteration. During each iteration, gradient boosting attempts to minimise the loss function.

Although gradient boosting works well with both categorical and continuous data, it sometimes overemphasises outliers and is computationally expensive. The xGBoost works by growing trees horizontally and is robust enough in handling heterogeneous data types and distributions. Moreover, xGBoost can efficiently handle sparse data patterns and also helps in handling overfitting [36]. LightGBM is a greedy implementation of the gradient boosting framework that uses gradient-based one-side sampling to grow trees vertically, which enables better identification of relationships between input and target features. However, LightGBMs are sensitive to outliers and can lead to overfitting if they are not handled properly [34,37].

Various techniques based on deep learning [32,33,34,35,36,38], support vector machines [32], ensemble models [38], and xGBoost [36] have been used in the literature, for estimating CO${}_{2}$ emissions from data gathered from vehicles.

In this paper, predicting CO${}_{2}$ emissions was based on ensemble learning techniques [39] that combine multiple regression models [40], which have been reported, in previous studies, to improve the prediction accuracy of machine learning algorithms [39]. Further improvements have also been achieved by incorporating strategies that combine various ensemble formation designs, such as stacked generalisation [40] and blending [41]. Stacked generalisation, proposed by Wolpert [42], is a procedure [43] that consists of several base learners (first level) and a meta learner (second level), where the outputs from the first level serve as inputs to the second level meta learners. The first-level regressors are fitted to the same training set that is used to prepare the inputs for the second-level regressor, which may lead to overfitting. However, the techniques proposed in [44] extend the standard stacking algorithm, using out-of-fold predictions, and produce the data from the first-level regressor, which serves as input for the second-level regressor, by splitting the dataset into *k* folds and, in every successive *k* rounds, k−1 folds are then utilised, to fit the first-level regressor. Furthermore, in each round, the first-level regressors are applied on the subset of the data that were initially excluded from the model fitting during each iteration. This allows the resulting predictions to be stacked and then forwarded as inputs to the second-level regressor, and then the first-level regressors are fitted on the entire dataset, to maximise the prediction accuracy. Blending is another ensemble approach that is derived from stacking [41], but differs by not using *k*-fold cross-validation to generate training data for the meta learner. However, it makes use of a one-holdout set, which allows a small portion of the data from the training set to be used for making predictions that can be used as inputs to the meta model.

**Figure 6:**
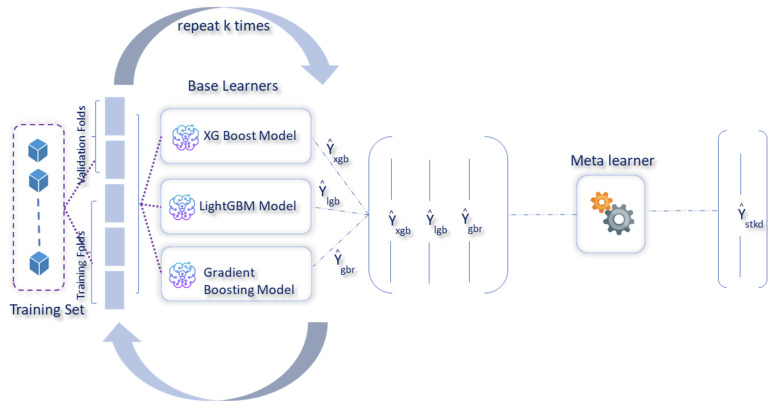
Stacked model, to estimate CO${}_{2}$ emissions.

**Figure 7:**
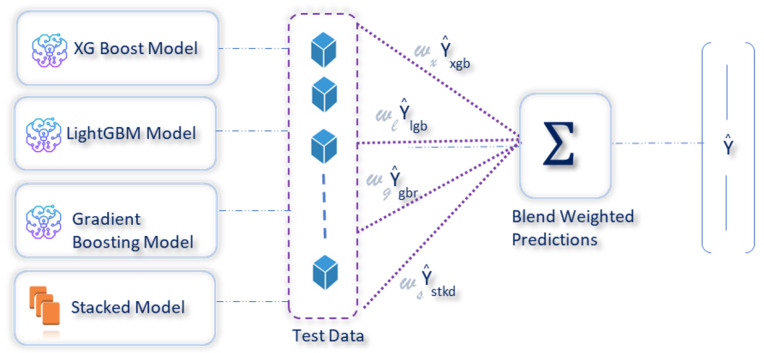
Weighted ensemble model, to estimate CO${}_{2}$ emissions.

In this paper, a weighted ensemble approach was used, which worked by assigning weights based on the individual model performance. Before the construction of the stacked model, the actual dataset was divided into training and test sets. The training set was then used to construct the stacked model, as shown in Figure 6. Following the identification of the best performing model from the individual baseline models during the stacking phase, weights were assigned to each of these models, with a higher weight to the stacked model followed by the best performing baseline model. The weighted predictions were then blended, to generate the final prediction. Using the model performances of the baseline models and the weighted ensemble (blended) model (Figure 7), weights of $w_{s}=0.4$, $w_{g}=0.3$, $w_{x}=0.15$, and $w_{l}=0.15$ were assigned, respectively, to the stacked, gradient boosting, xGBoost, and LightGBM models, following which, the weighted predictions were blended, to estimate the CO${}_{2}$ emissions.

### 5.1. Model Performance

**Figure 8:**
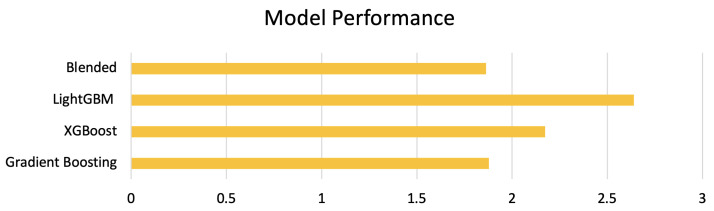
Model performance of blended, gradient boosting, xGBoost, and LightGBM baseline models.

In the weighted blended approach, the dataset is divided into *k* = 10 consecutive folds, with the shuffle parameter set to *True,* to avoid non-random assignment of the data points in the training and test sets. Each fold is used once for validation, while the rest of the k−1 folds then make up the training set. The root mean square error was used as a key performance measure for scoring. The mean square error returned by the cross-validation scoring function is always negative, and by selecting a negative mean squared error for scoring, the scoring function returns a positive score when the score has to be maximised and negates it when the score should be minimised. The baseline models were first trained individually, using the most common gradient-boosting tree-based machine learning algorithms in gradient boosting [35], xGBoost [36], and LightGBM [34], to identify the best-performing model baseline model. The stacked model was constructed using the best-performing baseline model as the meta model. The baseline models and the stacked model were used to construct the weighted ensemble model. The root mean square error (RMSE) metric was applied, to measure and compare the models’ performance. The RMSE metric indicated how far the predicted CO${}_{2}$ was from the average CO${}_{2}$ emissions. As seen in Figure 8, the RMSE score on the test dataset, using a higher weight gradient-boosting regressor as a meta regressor in the weighted ensemble model, was 1.8625, which was marginally better than the baseline gradient boosting model.

**Figure 9:**
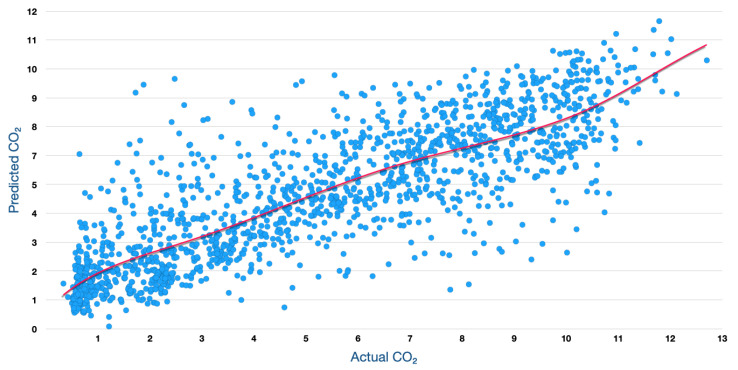
Actual vs. Predicted CO${}_{2}$ Emissions of the Weighted ensemble model from Test Data.

Figure 9 illustrates a visual assessment of how well the predictions made by a weighted ensemble model align with actual CO${}_{2}$ measurements. The plot has the model’s predictions on the vertical (y) axis and the observed CO${}_{2}$ values on the horizontal (x) axis. From this figure, it is evident that the data points cluster closely around a diagonal line, often referred to as the regression line. This line represents the scenario where predicted values perfectly match actual values.

In our context of predicting CO${}_{2}$ emissions, this closeness indicates that the model’s predictions are highly accurate. The tight distribution around the diagonal line suggests that the predicted CO${}_{2}$ values closely resemble the actual CO${}_{2}$ measurements. Furthermore, the vertical distance between each data point and the regression line, known as the prediction error or residual, appears small for most data points. Small prediction errors imply that the model’s predictions are very close to the actual values.

It is worth mentioning that the absence of significant patterns or systematic deviations from the diagonal line indicates that the model does not exhibit bias. This uniformity in predictions across the entire range of observed values is desirable in various applications.

In summary, the proximity of predicted CO${}_{2}$ values to the diagonal line reflects the excellent performance of the weighted ensemble model in providing accurate predictions. This outcome is highly favourable in data analysis, particularly in the context of IoT-based greenhouse gas sensing for real-time emissions monitoring applications, as it instils confidence in the model’s reliability for making predictions and drawing inferences.

### 5.2. Explaining CO${}_{2}$ Predictions

Lundberg et al. [45], have identified that interpretations and explanations of the predictions are as important as the accuracy of a predictive model.

When explaining CO${}_{2}$ predictions from the machine learning models, the predicted CO${}_{2}$, f(x), can be represented as the sum of its computed SHAP values and a fixed base value:

(1) $$f\left(x\right)=basevalue+sum\left(SHAP_{values}\right).$$

**Figure 10:**
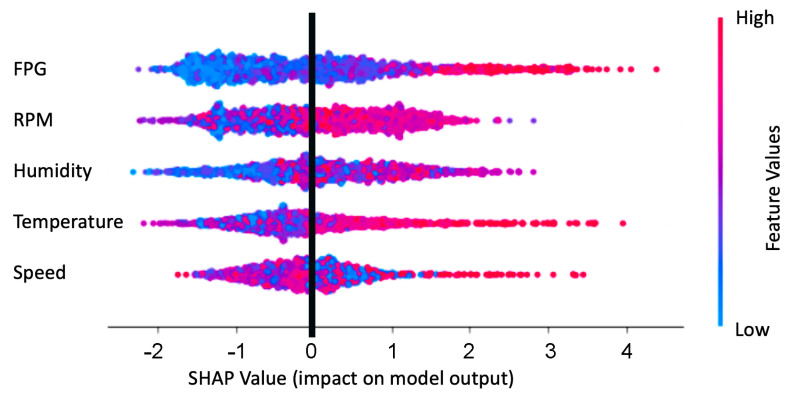
Relative feature importance and their relationship to the predicted CO${}_{2}$.

A SHAP (SHapley Additive exPlanations) [45] or Shapley value is the average marginal contribution of a feature value across all possible combinations, whereas the base value is the mean value of the target feature (CO${}_{2}$) in the dataset. The distribution of Shapley values helps in understanding the impact of FPG, RPM, humidity, temperature, and speed on CO${}_{2}$ predictions, both at local (individual) and global (population) levels. Local interoperability explains the individual predictions for each data instance, i.e., how the model arrived at a decision, in terms of the contributions of its input features, whereas global interoperability describes the expected behaviour of the machine learning model, with respect to the overall distribution of the values of all the input features. SHAP values are additive in nature, i.e., the SHAP values of FPG, RPM, humidity, temperature, and speed will always add up to the difference between the predicted CO${}_{2}$ from the baseline model and the current CO${}_{2}$ prediction that is being explained. Figure 10 provides information on the relationship between the features’ actual values and Shap values, i.e., the colour bar represents the actual values of the features (FPG, RPM, humidity, temperature, and speed) for each instance. A red-coloured dot indicates a relatively high value of the feature, and vice versa with a blue-coloured dot. We can see that higher values of FPG have positive SHAP values, i.e., the dots extending towards the right, forming the horizontal (0-axis) line are red, which indicates that FPG leads to higher CO${}_{2}$ predictions.

## 6. Discussion and Future Research

This paper describes the weighted ensemble approach, and the modelling results of this technique demonstrate that CO${}_{2}$ emissions can be predicted using data from onboard sensors from vehicles with considerably fewer features. The major contributions from this study can be summarised into (a) a weighted blended approach that uses data from onboard sensors to learn the CO${}_{2}$ emissions characteristics, (b) visual analysis, to identify and offset the lag from gas analyser measurements, and (c) performance comparison of the weighted ensemble approach to other popular tree-based approaches, such as LightGBM, xGBoost, gradient boosting, and stacked modelling approaches. The weighted ensemble approach works by assigning weights to each of the individual baseline models and the stacked model for predicting CO${}_{2}$ emissions.

In this approach, the highest weight was assigned to the stacked model, and then, among the baseline models, we assigned a higher weight to the best-performing baseline model, i.e., the gradient-boosting regressor, and then equally distributed the weight among the rest of the baseline models. However, it would be interesting to see the model performance on hyper-parameter tuned stacked and weighted ensemble models, instead of the baseline models, and also to compare the performance of the hyper-parameter tuned stacked model to the weighted ensemble model. Long et al. [46] have identified that geographical features (such as roads around mountainous regions), weather conditions (such as wind gusts, wind direction, and rainfall, etc.), and geographic terrains can impact CO${}_{2}$ emissions from vehicles. Furthermore, as our model does not generalise well, to accurately predict CO${}_{2}$ emissions at very low and high levels, it would be interesting to include contextual data in the model, describing the geographical features and traffic conditions, such as rush hours.

In Section 5.2, we also briefly explained the relative features importance and their relationships to the predicted CO${}_{2}$ emissions. In this paper, we have only focused on global interpretability, when explaining CO${}_{2}$ predictions [47]. However, in order to provide comprehensive explainability of the predicted CO${}_{2}$ emissions and the feature values, it is important to (a) relate the CO${}_{2}$ emissions with a variety of contextual information, such as geographical locations, and then explain the emissions, and (b) include local interpretability, when explaining the individual predictions.

Lastly, it is important to note that in application scenarios requiring substantial computational resources for real-time prediction of multiple transport vehicles, there are techniques like Map-Reduce [48,49], data approximation [50], contextualisation [51,52], and situation-aware computing [53] that can be employed, to achieve scalable real-time computation to meet the time-bound requirements [54,55].

## 7. Conclusions

Emissions reporting and sustainability have garnered significant global attention. However, a critical issue is the absence of a universally accepted method for quantifying emissions, especially in the case of Scope 3 emissions. This lack of standardisation, coupled with the complexities of logistics operations, poses substantial hurdles for businesses striving to comprehensively grasp and report their environmental impact. Traditionally, a common method for accurately capturing and monitoring gas emissions in logistics has centred on deploying gas sensors. Nonetheless, managing these sensors on moving vehicles, especially in the presence of varying road and weather conditions, poses a significant challenge. In this paper, we presented an innovative solution to addressing this challenge. Our approach leverages the IoT and AI, to eliminate reliance on gas sensors for real-time GHG emissions reporting, while accurately predicting CO${}_{2}$ emissions.

## Figures and Tables

**Table 1:** Gas analyser specifications.

Gases	CO (carbon monoxide) HC (hydrocarbons—hexane (gasoline), propane (LPG), or methane (CNG or LNG)) CO${}_{2}$ (carbon dioxide) O${}_{2}$ (oxygen) NO (NOx, nitric oxide)
Analysis Method	CO, HC, CO${}_{2}$: NDIR (non-dispersive infra-red) O${}_{2}$, NO: electro-chemical sensor
Ranges	CO: 0–10.00% HC (hexane and propane): 0–9999 ppm HC (methane) 0.000–5.000% CO${}_{2}$: 0–20.0% O${}_{2}$: 0.0–25.0% NO: 0–5000 ppm
Gas Sample Rate	350 mL/min typical. (flow-control pneumatic system).

**Table 2:** Starting and ending time of all IoT Sensors.

Sensor	Starting Time	Ending Time
OBDs	08:54:58	13:17:57
Gas Analyser	08:50:16	14:57:23
Temperature and humidity	08:52:26	14:58:34

**Table 3:** Feature set for estimating CO${}_{2}$ emissions.

Feature	Units of Measurement	Range of Feature Values
engine RPM	revolutions per minute	0–6000
fuel consumption (FPG)	grams per second	0–300
vehicle speed	kilometres per hour (km/h)	0–120
air temperature	Celsius (°C)	0–30
humidity	relative humidity in percentage (%)	0–100
